# Optimization of High-Density Fe-Au Nano-Arrays for Surface-Enhanced Raman Spectroscopy of Biological Samples

**DOI:** 10.3390/bios11060181

**Published:** 2021-06-05

**Authors:** Giovanni Marinaro, Maria Laura Coluccio, Francesco Gentile

**Affiliations:** 1 Institute of Process Engineering and Environmental Technology, Technische Universität Dresden, 01069 Dresden, Germany; 2 Nanotechnology Research Center, Department of Experimental and Clinical Medicine, University of Magna Graecia, 88100 Catanzaro, Italy; coluccio@unicz.it (M.L.C.); francesco.gentile@unicz.it (F.G.)

**Keywords:** plasmonic nanowires, molecular sensing, surface-enhanced Raman spectroscopy, porous alumina

## Abstract

The method of realizing nanostructures using porous alumina templates has attracted interest due to the precise geometry and cheap cost of nanofabrication. In this work, nanoporous alumina membranes were utilized to realize a forest of nanowires, providing a bottom-up nanofabrication method suitable for surface-enhanced Raman spectroscopy (SERS). Gold and iron were electroplated through the straight channels of the membrane. The resulting nanowires are, indeed, made of an active element for plasmonic resonance and SERS as the hexagonal distribution of the nanowires and the extreme high density of the nanowires allows to excite the plasmon and detect the Raman signal. The method to reduce the distance between pores and, consequently, the distance of the nanowires after electrodeposition is optimized here. Indeed, it has been predicted that the light intensity enhancement factor is up to 10^12^ when the gap is small than 10 nm. Measurements of Raman signal of thiol groups drying on the gold nanowires show that the performance of the device is improved. As the thiol group can be linked to proteins, the device has the potential of a biosensor for the detection of a few biomolecules. To assess the performance of the device and demonstrate its ability to analyze biological solutions, we used it as SERS substrates to examine solutions of IgG in low abundance ranges. The results of the test indicate that the sensor can convincingly detect biomolecules in physiologically relevant ranges.

## 1. Introduction

Over the past years, surface-enhanced Raman spectroscopy (SERS) has become a powerful tool allowing non-destructive, highly sensitive studies of molecules, chemicals or biological samples [1,2,3]. Further improvements of this technique could spur considerable progress in areas such as single-molecule sensing, early cancer detection and in situ analyte detection in microfluidics.

SERS takes advantage of highly packed sensitive nanostructures elements positioned in areas of few squared micrometers. Similar devices, fabricated thanks to recent advances in nanotechnology, enable the ultrasensitive, label-free detection of analytes. This detection can be enhanced, in turn, through integration with microfluidics that allows tight control over the volumes, flows and velocities of the biological liquids under examination. Microfluidics-assisted SERS has found applications in several fields, including biomedical engineering, proteomics, life science, and cellomics [4,5]. Notably, the combination of nanoscale devices and the manipulation of nano-liquids has enabled, among other things, the separation and identification of complex mixtures in very low abundance ranges [6,7].

While the technique achieves high sensitivity and ultra-low detection limit, nevertheless, the characteristics of sensitivity, precision, and selectivity have to be improved—this would make SERS devices suitable for the detection of biomarkers in complex solutions and biological fluids.

SERS devices amplify the Raman signal. Raman spectroscopy is a method that provides structural, chemical and conformational information about biomolecules such as proteins and DNA. In Raman spectroscopy, visible light and infrared radiation interact with a molecule, producing, as a result, a spectrum that describes the energy associated to the vibrational states of that molecule. Raman spectroscopy has a number of features, such as requiring minimal sample preparation and being label-free, non-destructive and non-invasive. The principal limitation of this spectroscopy is the extremely low Raman scattering cross-section (typically about 10^−24^ to 10^−27^ photons per events per molecule [8]) which is insufficient to characterize many biological systems, especially in low concentration ranges. Therefore, it is of primary importance to provide means to increase the signal intensity [9]. Among others, an efficient method for enhancing the Raman signal is to use plasmonic surfaces. Similar surfaces, typically made of a metal nanomaterial, manipulate and enhance the local electromagnetic (EM) field of several orders of magnitude. The EM field enhancement is the result of the collective, resonant oscillation of the electrons on the nano-metal’s surface [10]. The EM field increment has, as a consequence, the *surface enhancement* of the *Raman spectroscopy* signal, from which the acronym SERS is derived. The SERS effect provides access to otherwise unattainable information of biological systems, drugs, diluted analytes and biomolecules that are not detectable with conventional techniques of analysis [3].

The theoretical upper bound for SERS enhancement is 10^12^ [11]. Remarkably, since the SERS efficiency shows a very high sensitivity on the *geometry* of the substrate, in recent years, a variety of techniques have been developed to fabricate nanoscale structures with maximum resolution, maximum precision, and minimal tolerances [12,13,14,15,16,17]. Moreover, the design and fabrication methods for efficient SERS substrates should allow reliability and reproducibility over sufficient large areas to provide, at the same time, enhancement of the EM field, stability over time, and the ability to resist mechanical and environmental vibrations and noise. This leaves a lot of room for improvements in the design and fabrication of the final devices. Due to the topological requirements, the fabrication of SERS substrates involves nanotechnology techniques. Some representative examples are given in the following: Kattumenu et al. exploited nanorod-decorated nanowires to observe the Raman enhancement of thiolic molecules [18]. A super-hydrophobic surface made of micropillars was used to concentrate and detect a few molecules dissolved in a droplet [2]. Optical properties of a hexagonal array of metal nanopillars for plasmonic applications were investigated by Zhang et al. [19]. Menvod et al. functionalized graphene nanosheets with cationic poly (diallyldimethylammonium) (PDDA) and citrate-capped gold nanoparticles (AuNPs) for SERS bio-detection application [20]. Zhang et al. fabricated large-scale Au nanodisk arrays on Si substrate via x-ray interference for the detection of Rhodamine 6G as low as 10^−5^ mM [21]. Gentile et Al. dispersed silver nanoparticles into the pores of a superhydrophobic surfaces to guarantee superior SERS capabilities [1].

The present approach regards the use of plasmonic devices with large surface area and high-density hotspots whose increased detection efficacy is due to the strong plasmonic coupling of the nanowires. The sensitive device area is in the range of centimeters. While the single plasmonic elements can be made from 30 to 300 nm, their coupling distance can be adjusted between 3 nm and 20 nm. All these properties in the same device enable sensitive analysis of biological solutions, statistical significance, reliability, and repeatability. Moreover, the proposed technology is cheap and can be used in future translational biological medicine studies.

In this paper, the method has been used to detect Benzenedithiol molecules which were chemisorbed on the gold nanowire surface and, in another case, immunoglobuline IgG. The performance and the results of the biosensors will be presented in the next sections.

## 2. Materials and Methods

### 2.1. Nanowires Fabrication

**Figure 1:**
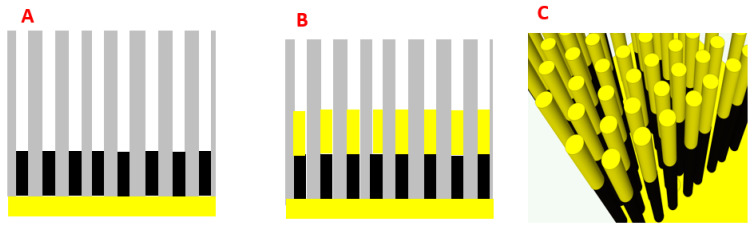
Sketch of Fe-Au electrodeposition through the pores of porous alumina: iron (**A**), addition of gold (**B**), and 3D illustration (**C**).

In order to efficiently and reproducibly fabricate NW-based substrates, the electrochemical deposition of NWs into nanoporous alumina templates was utilized (Figure 1). For a detailed description of the porous alumina fabrication, the protocol in a previous paper [8] was considered. The process involves two steps of anodization of a one-inch aluminum disk which create a thin layer of aluminum oxide with a highly ordered nanopore distribution. The first anodization step yields an alumina layer with poorly organized pores at the top, but high regularity at the bottom.

**Figure 2:**
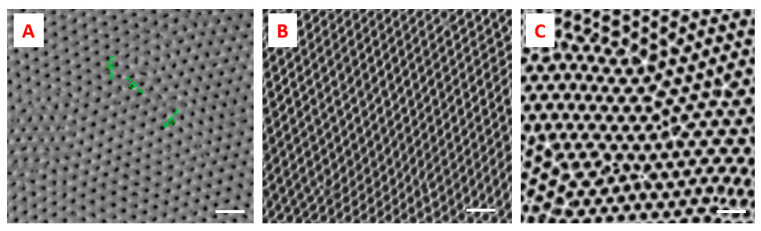
SEM micrographs. Porous alumina membranes with a distance between pores of 60 nm (**A**), 85 nm (**B**) and 95 nm (**C**). The white scale bar is 200 nm.

The pore distribution and size homogeneity are shown in Figure 2. The pores of alumina templates have a diameter of about 60 nm and are distributed in a hexagonal lattice with a constant distance of 40 nm. The electrolyte for the iron segment growth was realized with 6 g of iron sulfate heptahydrate (FeSO_4_·7H_2_O, Sigma-Aldrich), 1 g of boric acid (H_3_BO_3_, Sigma-Aldrich) and 1 g of ascorbic acid (C_6_H_8_O_6_, Sigma-Aldrich). Boric acid was added to improve the purity of iron while ascorbic acid was added to adjust the pH to 3.

The electrolyte for the gold segment growth was prepared with 0.1 potassium dicyanoaurate (KAu(CN)_2_, Sigma-Aldrich, St. Louis, MO, USA) and 4 g of boric acid (H_3_BO_3_, Sigma-Aldrich). Boric acid was added in order to adjust the pH and work in safe acidic conditions. In order to realize three different sizes of the pores, and consequently, three different aspect ratios of nanowires, the membranes were dipped in 5% H_3_PO_4_ in water (*w*/*w*) for different lengths of time. Porous alumina was then etched away in an acidic chrome solution (CrO_3_/H_3_PO_4_ in water) at 40 °C overnight (Figure S1) in order to obtain free-standing nanowires.

Removal of the alumina reveals highly ordered features on the surface of the Al substrate, which facilitates the growth of ordered pores upon the second anodization step. The pores obtained with the second anodization are straight channels arranged in a hexagonal pattern. The diameter of pores and the distance between them can be tuned by controlling the parameters of anodization, while their length depends on the anodization duration. One of the main challenges in using porous membranes for SERS is to reduce the gap between the active elements of the nanostructure because, based on numerical predictions, the light intensity shows the best enhancement factor (up to 10^12^) when the gap is smaller than 10 nm.

A way to address the problem to a solution is to gently dip porous alumina in a diluted phosphoric acid solution in order to widen the pores and, consequently, reduce the inter-distance to a few 10s of nanometers. Simulations have already predicted that nanostructures obtained by this method are suitable for SERS devices [8].

After removing the backlayer substrate of alumina with an etching solution, an electric contact was made using a PECVD by depositing a thin layer of 20 nm of ITO (Indium Tin Oxide) on the surface of the porous alumina membrane. The electrolyte for the iron segment growth was realized with 6 g of iron sulfate heptahydrate (FeSO_4_·7H2O, Sigma-Aldrich), 1 g of boric acid (H_3_BO_3_, Sigma-Aldrich) and 1 g of ascorbic acid (C_6_H_8_O_6_, Sigma-Aldrich). Boric acid was added to improve the purity of the iron while ascorbic acid was added to adjust the pH to 3. The electrolyte for the gold segment growth was prepared with 0.1 potassium dicyanoaurate (KAu(CN)_2_, Sigma-Aldrich) and 4 g of boric acid (H_3_BO_3_, Sigma-Aldrich). Boric acid was added in order to adjust the pH and work in safe acidic conditions.

### 2.2. SEM Analysis

SEM analysis of the plasmonic nanowires was conducted with a Zeiss GeminiSEM 500 at Dresden Center for Nanoanalysis (DCN), TU Dresden. The samples were already conductive, so there was no need to sputter gold. The samples were fixed on a stub with a long pin and then mounted on a carousel 9 × 9 mm sample holder. In order to fix the samples, a small amount of silver paint was applied between the edge of the aluminum disc and the stub. A further copper lever was screwed in order to secure the sample on the stub. Several images of metal nanowires were acquired in high vacuum mode at 5 kV, a magnification of 300,000 and a working distance of about 3 mm with an InLens Detector (ZEISS) for secondary electrons. In order to reduce the noise, a frame integration (N = 14) was performed. With this setup, every frame was scanned and averaged 14 times.

### 2.3. Light Transmission Measurements

**Figure 3:**
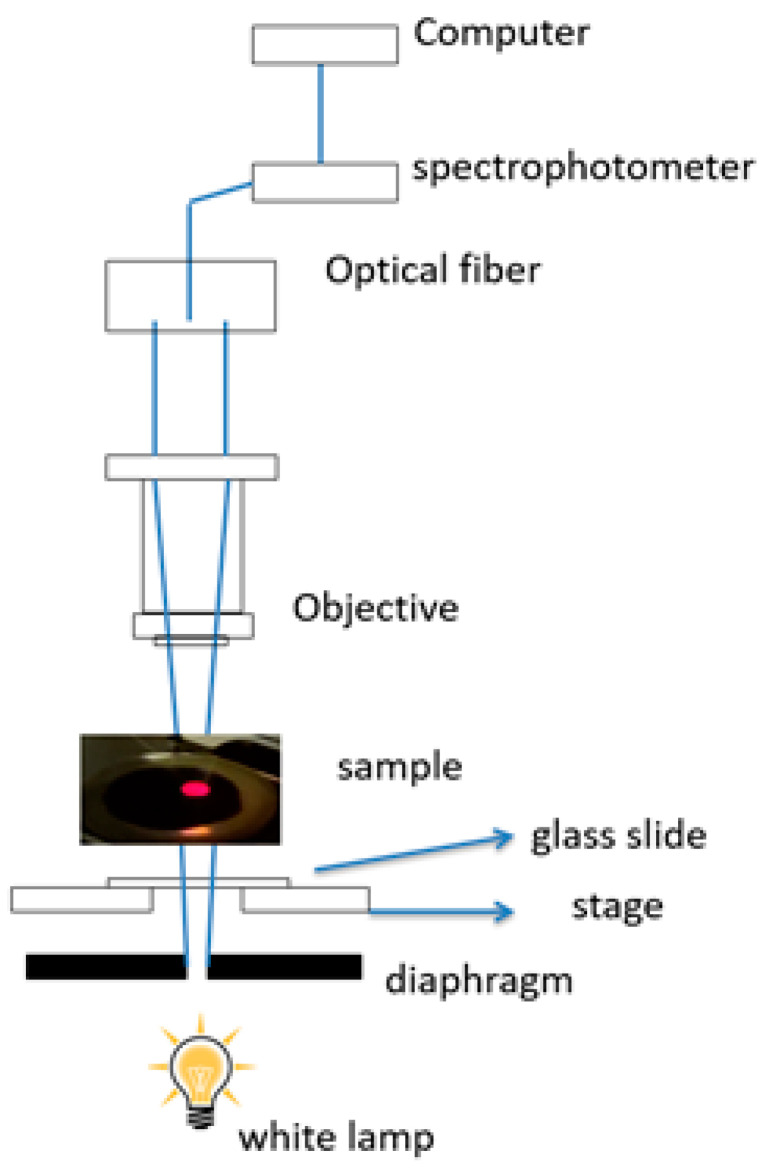
Ray diagram of light transmission setup. The onset shows the plasmonic device illuminated from the backside.

A Nikon Eclipse Ni with an integrated Thor spectrometer (a ray diagram is shown in Figure 3) was used to measure the wavelength band of white light in transmission.

To do so, a variation in the fabrication step was realized: an ITO nanofilm was sputtered to the back of the alumina membrane as a conductive layer for the electrodeposition instead of sputtering gold. ITO is a material that is conductive and transparent at the same time. This guaranteed the electrodeposition of nanowires through the pore channels and the ability to perform optical light measurements.

### 2.4. Raman Spectroscopy

A Renishaw InVia Raman spectrometer with a 1200 line/mm grating for the SERS measurements was used for the measurement of the Raman signals. The samples were excited by 830 nm laser line in backscattering configuration through 100× objective (NA = 0.9) using the respective edge filters to stop the laser lines. The scattering was collected in the range of 200 to 2000 cm^−1^. The spectra were analyzed with WiRE 3. Benzenedithiol 1,4 molecules were deposited on the substrates by immersion in solution and subsequent rinsing in MilliQ water. A droplet of about 10^−1^ mM of benzedithiol 1,4 in ethanol was gently deposited on the surface of the device with a pipette and allowed to dry for about one hour in order to stabilize the disulphuric links of thiols with the gold nanowires. SERS spectrum of Benzenedithiol 1,4 was excited with a laser power of 2 mW. Further investigations were carried out with an HR 800 Raman spectrometer with a micro-Raman spectral acquisition images. The samples were excited with a 795 nm laser line through a 100× objective (NA = 0.9). The spectra were exported as text files and analyzed with an in-house script written and run in Matlab (R2017b MathWorks).

### 2.5. SERS Analysis of IgG

A drop of immunoglobulin (IgG) at 0.44 mg/mL was positioned on the substrate and left to dry. The Raman spectra of IgG adsorbed on the substrate were collected by an InVia Raman spectrometer with a 1200 line/mm grating, equipped with a 100× optical microscope objective. Samples were excited by an 830 nm laser line, setting the laser power at 1.6 mW. The Raman signals were recorded on maps of different sizes in a spectral range of 800 to 1800 cm^−1^ and an integration time of 2 s for each point. The map spectra were analyzed with WiRE 3 and elaborated with Wolfram Mathematica (The Wolfram Centre, Oxford, United Kingdom), analyzing each map on the basis of characteristic peak intensities (1250, 1330 and 1450 cm^−1^). Individual Raman spectra were baseline corrected using a polynomial passing through at least eight points uniformly distributed in the spectral range. Then, spectra were rescaled in the intensity direction using min–max normalization, whereby the minimum value of a spectrum intensity is transformed into a 0, the maximum value is transformed into a 1, and every other value is transformed into a decimal between 0 and 1 [22].

## 3. Results—SERS Device

### 3.1. Characterization of Nanowires

**Figure 4:**
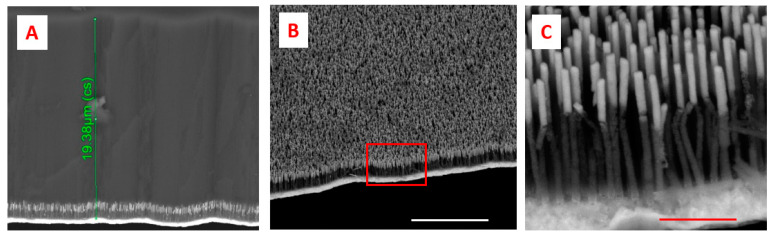
SEM micrographs. (**A**). Porous alumina with electroplated nanowires at the bottom near the metal contact. The thickness of the membrane is roughly 20 μm, as indicated from the green line. (**B**). Nanowires after alumina etch( scale bar 5 μm). (**C**). Magnified region marked as red square in Figure 3B; the scale bar is 500 nm.

The SERS device, realized according to the description in the previous session, was characterized both with SEM during different steps of fabrication and with a spectrometer connected to an optical microscope, as described in the materials and methods session. Figure 2A shows the alumina membrane after the two steps of anodization. Figure 2B shows the template after 100 min of the widening process, which allows 15 nm of pore distance; Figure 2C shows the template after 120 min of the widening process, with a homogeneous pore distance of 5 nm. An ITO nanofilm was sputtered to the back of the alumina membrane as a conductive layer for the electrodeposition. Porous templates were then used to grow metal nanowires through the pores after electrodeposition. The electrodeposition Fe-Au nanowires form a composite material together with the alumina template near the bottom (Figure 4A), and after the removal of the alumina, free-standing Au-Fe nanowires were finally obtained(Figure 4B,C).

**Figure 5:**
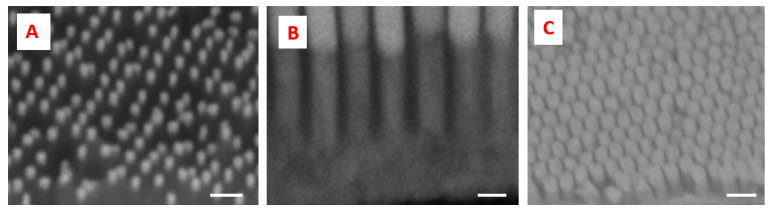
SEM micrographs. Fe-Au nanowires with distance between wires 40 nm (**A**), scale bar = 200 nm, 15 nm (**B**), scale bar = 90 nm and 5 nm (**C**), scale bar = 180 nm.

Since three different typologies of porous alumina templates based on pore diameter were realized, an increasing plating time was applied for small, medium and large pores in order to fill the different hollow volumes of the nanoporous material and ensure about the same height of nanowires. Figure 5A shows the top of the gold nanorods obtained with a porous alumina template without the widening process. Figure 5B,C shows Au-Fe nanowires with a distance, respectively, of 15 and 5 nm.

Transmission spectra of the nanowires was acquired and then convoluted by using a Savitzky–Golay filter. The peaks at 747 nm, 820 nm and 810 nm were transmitted with higher intensity by the nanowires with smaller distance (Figure S2), suggesting that the plasmonic resonance of the gold nanowires is higher when they are excited with an infrared laser line. For this reason, Raman spectroscopy was conducted with a laser line of 833 nm.

### 3.2. Detection of Benzenedithiol and IgG Solution Dried on the Biosensor

**Figure 6:**
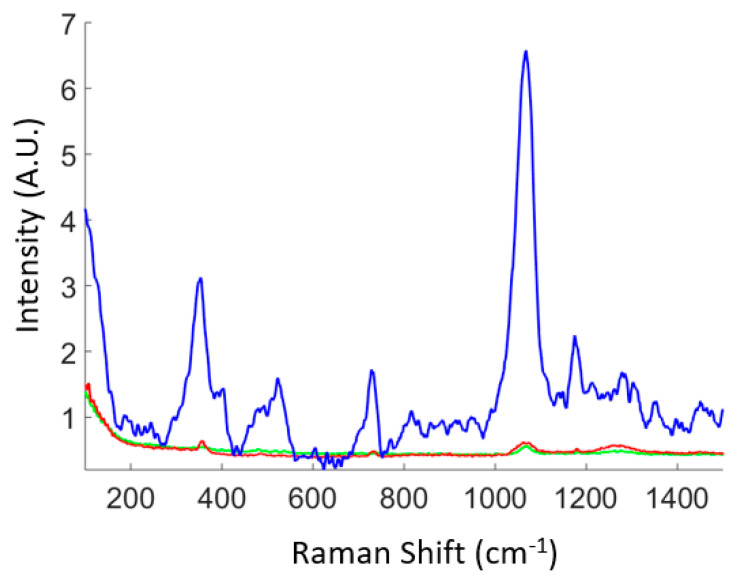
Raman spectra of Benzenedithiol 1,4 chemiosorbed on Au nanowires with large distance (green line), nanowires with middle distance (red), and nanowires with small distance between nanowires (blue line).

Free-standing Fe-Au nanowires were fabricated using the electrochemical method after two consecutive steps of electrodeposition. The SERS spectrum of Benzenedithiol 1,4 is shown in Figure 6. The performance of the device with 5 nm gap nanowires, as shown in Figure 6, is much better than the other cases when the distance is larger. The estimation of the signal intensity is of the order of 10^4^ with respect to the thicker nanowire device. The signal of Benzenedithiol for the small gap spacing was measured at different points by mapping a region of the biosensor. The signal in Figure 6 (blue curve) is extracted from one point of the map. We repeated the spectra acquisition several times at that point and did not observe relevant changes. We mapped the other surfaces (with higher gaps between nanowires) and detected a poor signal.

As predicted by simulations in a previous work, the SERS signal of Benzenedithiol 1,4 shows a higher intensity due to the fact that the dipoles of gold nanorods on the head of nanowires, realized in this work, have, in the infrared region, a better electric-field enhancement, which was estimated to have a factor of 10^4^. In Figure S4 of the Supporting Information, we report the SERS signal coming from Benzenedithiol (BDT) measured by the nanowire sensor device with three different configurations (large, middle, small gap) compared to the Raman spectrum of BDT acquired over a flat non-SERS substrate (flat silicon surface). In the image, all spectra are individually normalized to the maximum peak in the spectral range. Remarkably, the SERS and Raman signal are significantly different, proving the selective adsorption and enhanced Raman activity of BDT over the SERS nanowires device, compared to the spontaneous Raman scattering of the molecule in standard conditions.

**Figure 7:**
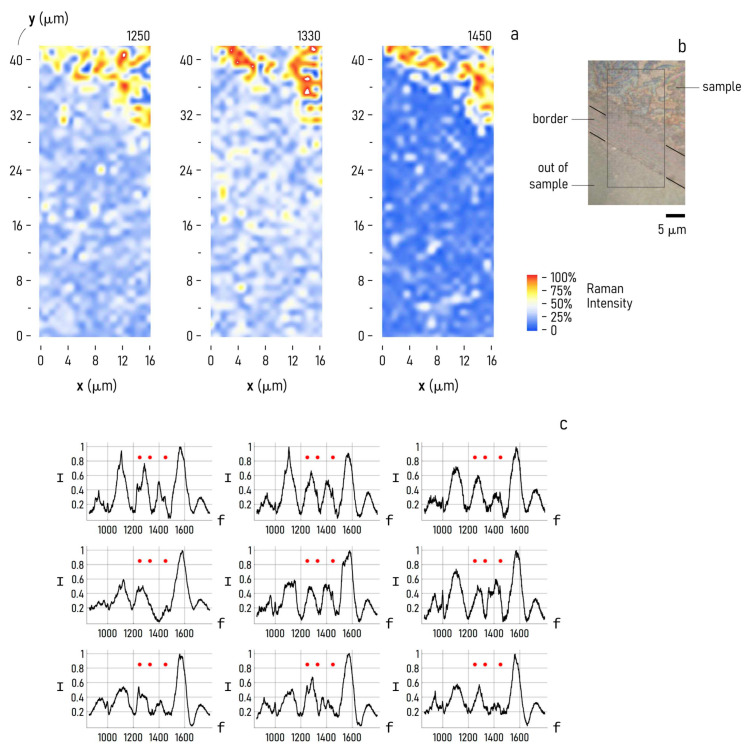
SERS maps of IgG positioned on the sensor device, acquired at 1250, 1330 and 1450 cm^−1^, respectively (**a**). Optical image of the sample drop after evaporation on the device, and the region of the sample surface interrogated through SERS analysis (**b**). Collection of nine spectra randomly sampled from the SERS maps, where the characteristic peaks of IgG have been highlighted (**c**).

To further assess the performance of the device, we used the biosensor to examine a solution of antibodies in low abundance ranges. Upon casting a sample drop containing IgG with an initial concentration of 0.44 mg/mL on the sensor surface, we waited until the complete evaporation of the solvent and analyzed the residual using the Raman setup described in the methods of the paper. The Raman maps reported in Figure 7 were collected over a region of the sensor device partially coated with the sample drop. The maps in Figure 7a report the normalized Raman spectrum intensity measured at 1250 cm^−1^, 1330 cm^−1^ and 1450 cm^−1^, respectively. Notably, the signal distribution in the maps matches with very high accuracy with the originating layout of the sample drop on the device (Figure 7b). The signal is high within the contact area of the sample with the sensor surface, then it sharply falls to nearly zero, moving away from the biological sample towards the free sensor surface. The very high correspondence between the expected spatial distribution of the biological sample with the Raman maps indicates that the device and the entire method is effective in performing the analysis of biological solutions. The spatial frequencies of 1250 cm^−1^, 1330 cm^−1^ and 1450 cm^−1^ that we have used as a reference in the analysis are central lines where the IgG vibrational activity is preferentially expressed. They represent the fingerprint of IgG.

IgG molecules are characterized by a significant percentage (47%) of β-sheet conformation and only 7% of α-helix [23]. The β-sheet secondary structure is identified by the amide I broad band at 1673 cm^−1^ and by the amide III region with a slight band at around 1243 cm^−1^. In the amide III region, the band at 1336 cm^−1^ is also observable, evidencing the α-helix secondary structure portion [24]. The CH2 deformation (ρCH2) band is observed at around 1450 cm^−1^, associated with the protein structures. Other minor peaks are related to amino-acidic residues (e.g., phenylalanine at 1004 cm^−1^) and to the backbone skeletal vC–C vibration bands around 1030 to 1170 cm^−1^ [25].

In Figure 7c, we report a number of Raman spectra extracted from the full-field Raman maps described above. Each of those spectra, randomly sampled from the maps, exhibit characteristic peaks at no less than one of the following frequencies: 1250 cm^−1^, 1330 cm^−1^ and 1450 cm^−1^. Remarkably, above roughly 1500 cm^−1^, the Raman spectra in the grid seem to convey no or little information about the biological sample, perhaps because environmental or instrumental noise obscure the sample emission. Below the 1500 cm^−1^ limit, the frequency content of the signal is consistent with the biological sample being IgGs.

The Raman spectra that we have reported in Figure 7c are representative examples of a larger set of data, all having the property of showing a very high Raman signal at 1250 cm^−1^, 1330 cm^−1^, 1450 cm^−1^, or a combination of these three frequencies. This is illustrated from the Raman maps reported in Figure 7a, where the signal calculated in correspondence with those reference values shows minimal irregularity. To demonstrate repeatability more convincingly, we report in Figure S3 of the Supporting Information section the complete set of Raman spectra acquired over the active area of the sensor device.

## 4. Discussions

The SERS signal coming from molecules in close proximity to a plasmonic nanomaterial shows a very high sensitivity to the distance of the molecules to the surface and the molecule orientation, which are parameters that are not or are minimally influenced by the operator during the measurement. As a result, for its nature, the SERS analysis of a compound and corresponding Raman spectra show poor reproducibility and repeatability. This limits the use of the technique as a quantitative method of analysis of biological systems. Nevertheless, while not perfectly identical, the Raman spectra that we have shown in Figure 7c all show a pattern similarity. Centered either at 1250 cm^−1^, 1330 cm^−1^ or 1450 cm^−1^, the spectra have a peak that is characteristic of IgG, as reflected by the maps reported in part of the same figure, where the Raman intensity calculated for those peaks shows very high uniformity over the sample sensing area.

To understand the clinical implications of the results, it is useful to put the IgG concentration of 0.44 mg/mL that we have used in this work in context. The typical values of IgG normally found in adults fall in the 7 to 15 mg/mL interval, called the reference range [26], which are between 15 and 30 times higher than our study’s sample concentration. Thus, even without testing the device with ultra-low concentrated solutions, the results of the work indicate that this biosensor is suitable to detect IgG fluctuations downward or upward relative to the reference range. Upward oscillations (high levels of IgG) may be indicative of pathological states including chronic infection, such as HIV, multiple myeloma, chronic hepatitis, and multiple sclerosis. On the other hand, downward oscillations (low levels of IgG) occur, such as, for example, in macroglobulinemia; in some types of leukemia; and in nephrotic syndrome, a type of kidney damage. Further to this end, in a more sophisticated evolution of the device that will be developed over time, the gold sensor surface will be functionalized with antibodies [27] or aptamers [28] for the selective capture of biomarkers. This sensing device will achieve the recognition of antigens in complex mixtures in very low abundance ranges, combining the characteristics of low cost, high resolution, high sensitivity, and selectivity.

## 5. Conclusions

In the present work, a method of SERS device nanofabrication using alumina template was optimized for molecular sensing. To do so, alumina templates were fabricated according to two anodization steps. The SERS device, realized according to the description in the previous sessions, was characterized geometrically and optically with SEM during different steps of fabrication and with a spectrometer. Porous templates were used to grow metal nanowires through the pores after electrodeposition. A series of SERS measurements at 830 nm using a forest of nanowires, organized in a hexagonal lattice, with a gap of only 5 nm to detect the Raman signal of chemisorbed benzenedithiol 1,4 shows a large increase in the local intensity with respect to the forest of nanowires with larger distance. As reported in a previous paper [8], the enhancement factor of the Raman signal for this device is of the order of 10^4^. As the thiol group can be linked to many biomolecules of interest such as proteins, the device has the potential to be used as a biosensor for the detection of a few biomolecules in different fields such as microfluidics, proteomics and optoelectronics. Further analysis of solutions of biomedical interest—i.e., IgG—in low abundance ranges confirm the ability of the device to detect biomolecules with potential applications in the treatment and diagnosis of diseases.

## Data Availability

The data that support the findings of this study are available from the corresponding authors upon reasonable request.

## Supplementary Materials

The following are available online at https://www.mdpi.com/article/10.3390/bios11060181/s1, Figure S1: Etching process of porous alumina. The samples are upside down and floating on chrome solution which is kept at 40 °C. Figure S2: Etching process of porous alumina. The samples are upside down and floating on chrome solution which is kept at 40 °C. Figure S3: Complete set of Raman spectra acquired over the active area of the sensor device. Figure S4: SERS signal coming from Benzenedithiol (BDT) measured by the nanowires sensor device with three different configurations (big, middle, small gap) compared to the Raman spectrum of BDT acquired over a flat non-SERS substrate (flat Silicon surface). In the image, all spectra are individually normalized to the maximum peak in the spectral range.
