# Supplementary material for: Optimization of High-Density Fe-Au Nano-Arrays for Surface-Enhanced Raman Spectroscopy of Biological Samples
Source: Biosensors (Basel). 2021 Jun 5;11(6):181. doi: 10.3390/bios11060181 (PMC8229969; doi:10.3390/bios11060181)
Supplement: Supplementary file 1 [file biosensors-11-00181-s001.zip › biosensors-1227951-supplementary.pdf]

Optimization of High-Density Fe-Au Nano-Arrays for Surface-Enhanced Raman Spectroscopy of Biological Samples

Giovanni Marinaro [1], Maria Laura Coluccio [2], Francesco Gentile [2]

[1] Institute of Process Engineering, Technische Universität Dresden, 01069 Dresden, Germany

[2] Nanotechnology Research Center, Department of Experimental and Clinical Medicine, University of Magna Graecia, 88100 Catanzaro, Italy

Corresponding author: giovanni.marinaro@kaust.edu.sa

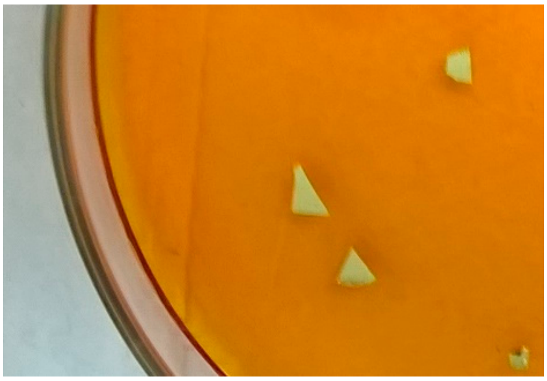

Figure S1. Etching process of porous alumina. The samples are upside down and floating on chrome solution which is kept at 40 °C.

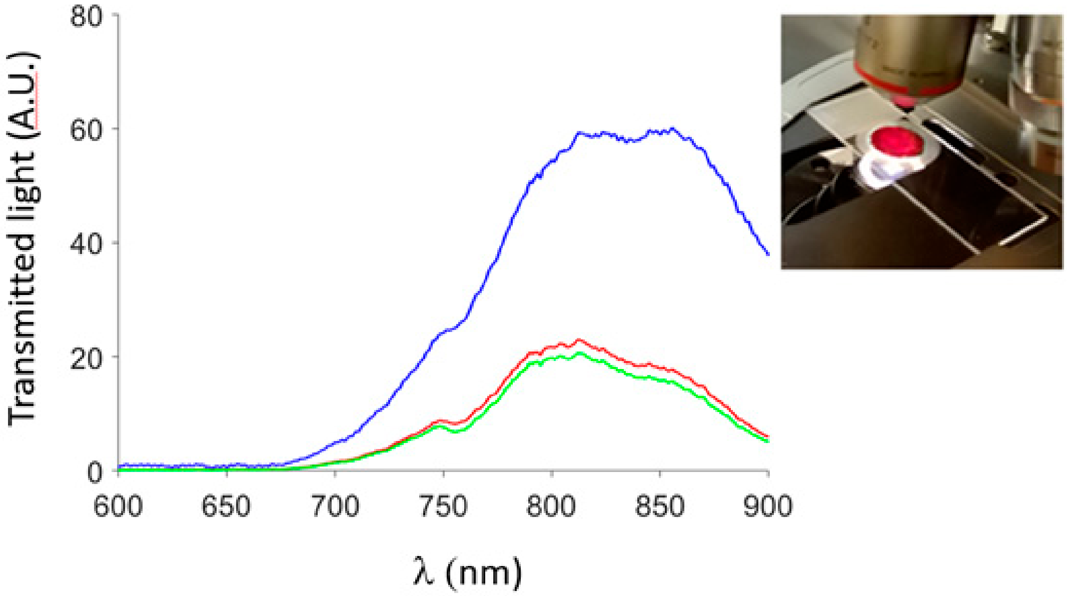

Figure S2. Etching process of porous alumina. The samples are upside down and floating on chrome solution which is kept at 40 °C.

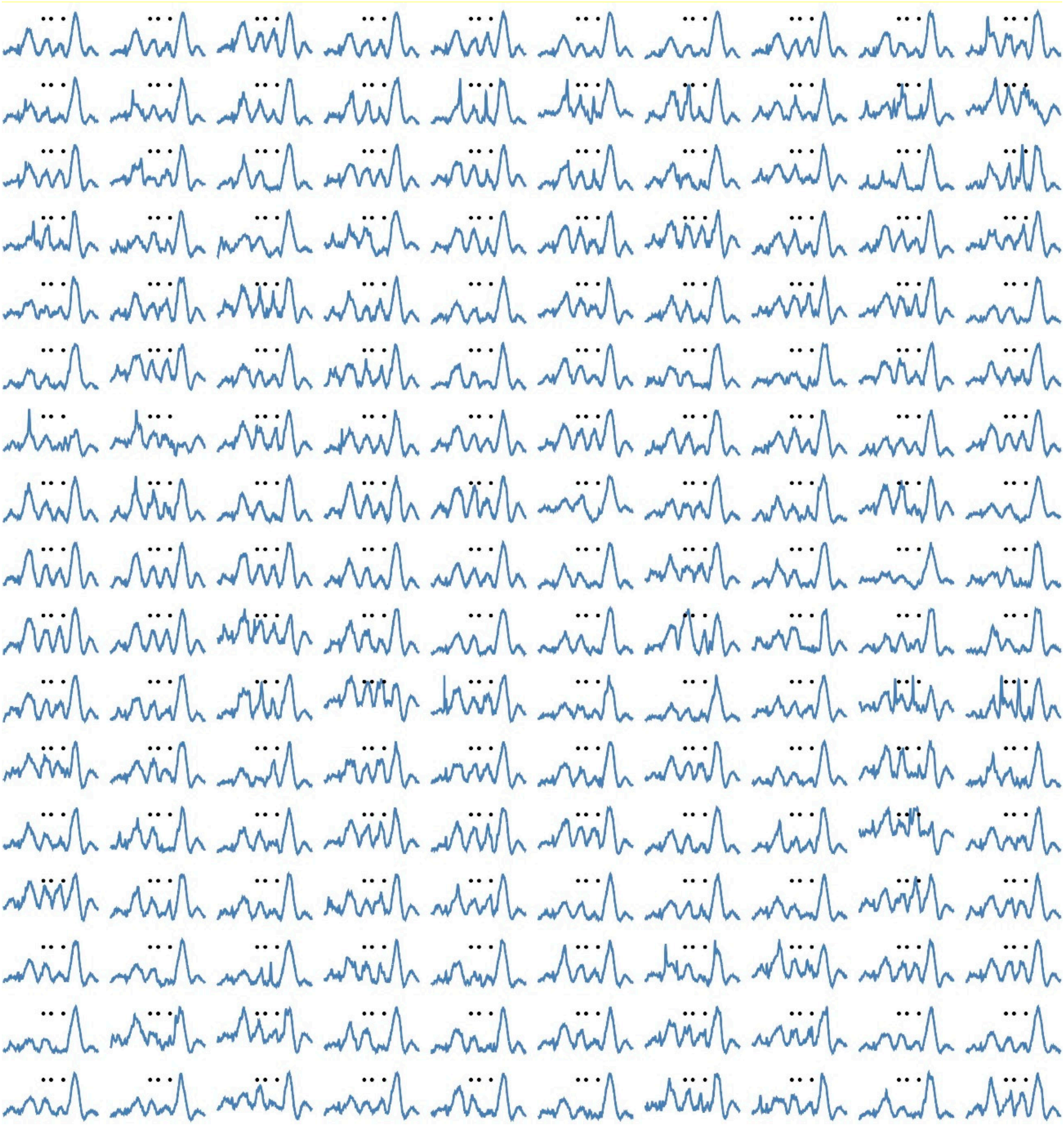

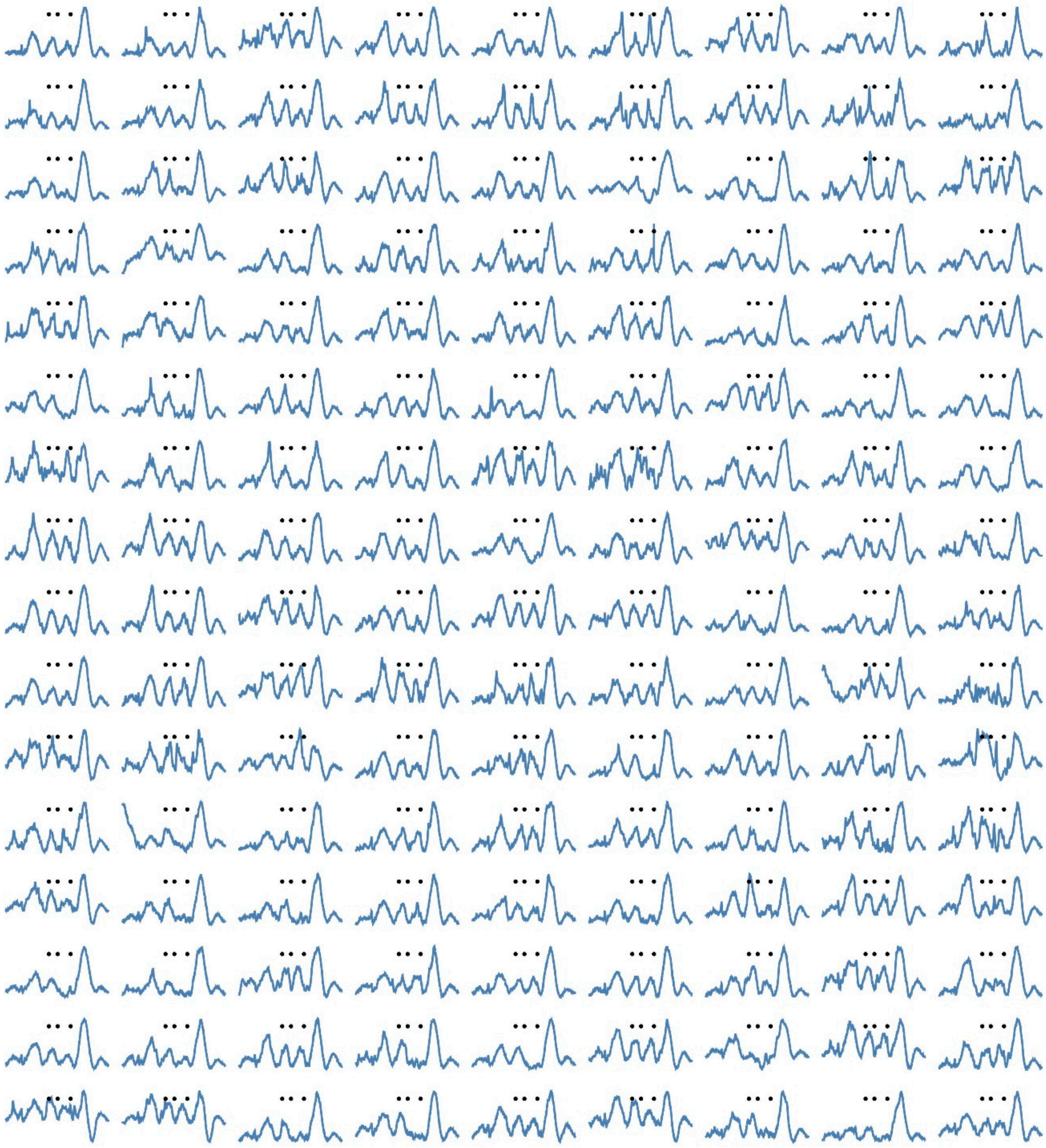

Figure S3. Complete set of Raman spectra acquired over the active area of the sensor device.

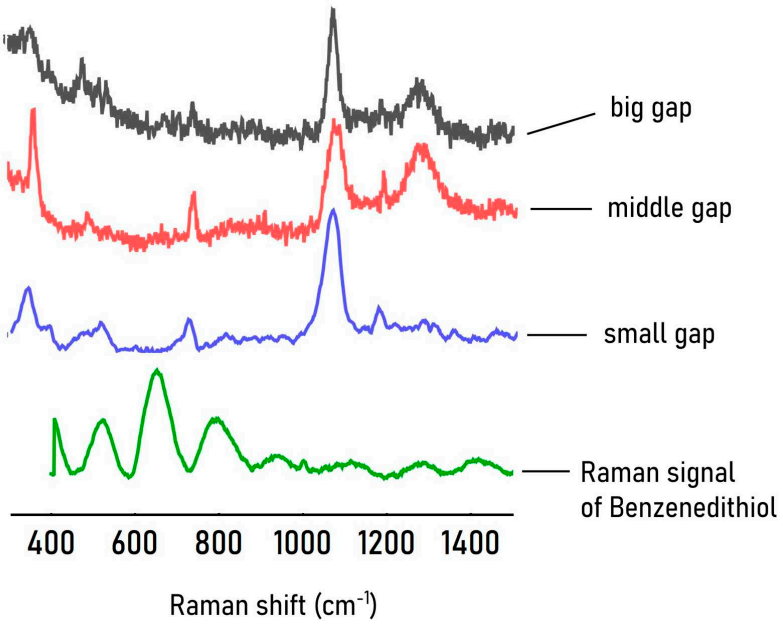

Figure S4. SERS signal coming from Benzenedithiol (BDT) measured by the nanowires sensor device with three different configurations (big, middle, small gap) compared to the Raman spectrum of BDT acquired over a flat non-SERS substrate (flat Silicon surface). In the image, all spectra are individually normalized to the maximum peak in the spectral range.
